# Multiple sclerosis in *LRRK2* G2019S Parkinson’s disease and isolated nigral degeneration in a homozygous variant carrier

**DOI:** 10.3389/fneur.2024.1450654

**Published:** 2024-08-08

**Authors:** Adina Wise, Roberto A. Ortega, Deborah Raymond, Alessandra Cervera, Emma Thorn, Katherine Leaver, David S. Russell, Susan B. Bressman, John F. Crary, Rachel Saunders-Pullman

**Affiliations:** ^1^Department of Neurology, Mount Sinai Beth Israel Medical Center, New York, NY, United States; ^2^Department of Neurology, Icahn School of Medicine at Mount Sinai, New York, NY, United States; ^3^Departments of Pathology, Neuroscience, and Artificial Intelligence & Human Health, Icahn School of Medicine at Mount Sinai, New York, NY, United States; ^4^Neuropathology Brain Bank & Research CoRE, Friedman Brain Institute, Icahn School of Medicine at Mount Sinai, New York, NY, United States; ^5^Ronald M. Loeb Center for Alzheimer’s Disease, Icahn School of Medicine at Mount Sinai, New York, NY, United States; ^6^Institute for Neurodegenerative Disorders, New Haven, CT, United States

**Keywords:** multiple sclerosis, Parkinson’s disease, LRRK2, genetic, genetic risk, Parkinson’s genes, immunology & inflammation, autoimmune diseases

## Abstract

**Background:**

*LRRK2* variants have been associated with immune dysregulation as well as immune-related disorders such as IBD. A possible relationship between multiple sclerosis (MS) and *LRRK2* PD has also been suggested. Further, neuropathologic studies of homozygous *LRRK2* G2019S carriers with Parkinson’s disease (PD) are rare, and there are no systematic reports of clinical features in those cases.

**Methods:**

We investigated the co-occurrence of PD and MS in our research cohort and report on two cases of MS in *LRRK2* PD as well as neuropathological findings for one.

**Results:**

MS preceded PD in 1.4% (2/138) of participants with *LRRK2* G2019S variants, and in none (0/638) with idiopathic PD (*p* = 0.03). One case with MS and PD was a *LRRK2* G2019S homozygous carrier, and neuropathology showed evidence of substantia nigra pars compacta degeneration and pallor without Lewy deposition, as well as multiple white matter lesions consistent with MS-related demyelination.

**Discussion:**

The increased prevalence of MS in *LRRK2* PD further supports an important role for immune function for *LRRK2* PD. This co-occurrence, while rare, suggests that MS may be an expression of the *LRRK2* G2019S variant that includes both MS and PD, with MS predating features diagnostic of PD. The neuropathology suggests that the MS-related effects occurred independent of synuclein deposition. Importantly, and in addition, the neuropathological results not only support the MS diagnosis, but provide further evidence that Lewy body pathology may be absent even in homozygote *LRRK2* carriers.

## Introduction

An immune role for *LRRK2* in mediating PD has been suggested (1), and the question of increased frequency of concomitant multiple sclerosis and *LRRK2* G2019S-related PD (*LRRK2* PD) has been raised (2). Homozygous *LRRK2*-related Parkinson disease (PD) has been reported and does not appear motorically worse than heterozygous *LRRK2* PD (3), however, descriptions of non-motor features and neuropathology in these cases are limited (4).

## Methods

Participants were part of genetic studies of PD at Mount Sinai Beth Israel/Mount Sinai, and screening for *LRRK2* G2019S and eleven *GBA* variants was performed as previously reported (5). Twelve individuals with both *LRRK2* G2019S and *GBA1* variants were excluded from the analysis. History of multiple sclerosis was ascertained by patient report and prevalence of MS was compared between *LRRK2* PD and non-*LRRK2* PD groups. The non-*LRRK2* group was comprised of 142 individuals with *GBA* PD and 496 individuals with idiopathic PD. In the case with neuropathology, olfaction was measured by University of Pennsylvania Smell Identification Test (UPSIT), striatal dopamine transporter density assessment using iodine-123–labeled 2-β-carboxymethoxy-3-β-(4-iodophenyl)tropane single photon emission-computed tomography (β-CIT SPECT), and transcranial sonography (6, 7) was performed. The Mount Sinai IRB approved both the overall studies (AAAE0748 and AAAF3108), and the neuropathological protocols; next-of-kin provided consent for brain donation. Histopathological workup consisted of a comprehensive neuroanatomical sampling assessed according to standard protocols as previously described (8). Routine (hematoxylin & eosin, Luxol fast blue, and Bielschowsky silver) and a battery of immunohistochemical (IHC) stains for phospho-tau (AT8, 1:1000, Invitrogen), β-amyloid (4G8, 1:8000, BioLegend), TAR DNA-binding protein 43 (TDP-43, 1:5000, Proteintech), α-synuclein (LB509, 1:3000, Abcam), and neurofilament (SMI-32, 1:1000, Biolegend).

## Results

MS was present and preceded PD in 1.4% (2/138) of participants with *LRRK2* G2019S variants. This was increased relative to the non-*LRRK2* PD group in which no participants had an MS diagnosis that preceded the onset of PD (*p* = 0.03). In the subset of women, 2.9% (2/69) of *LRRK2* PD participants had preceding diagnoses of MS (*p* = 0.05).

**Figure 1:**
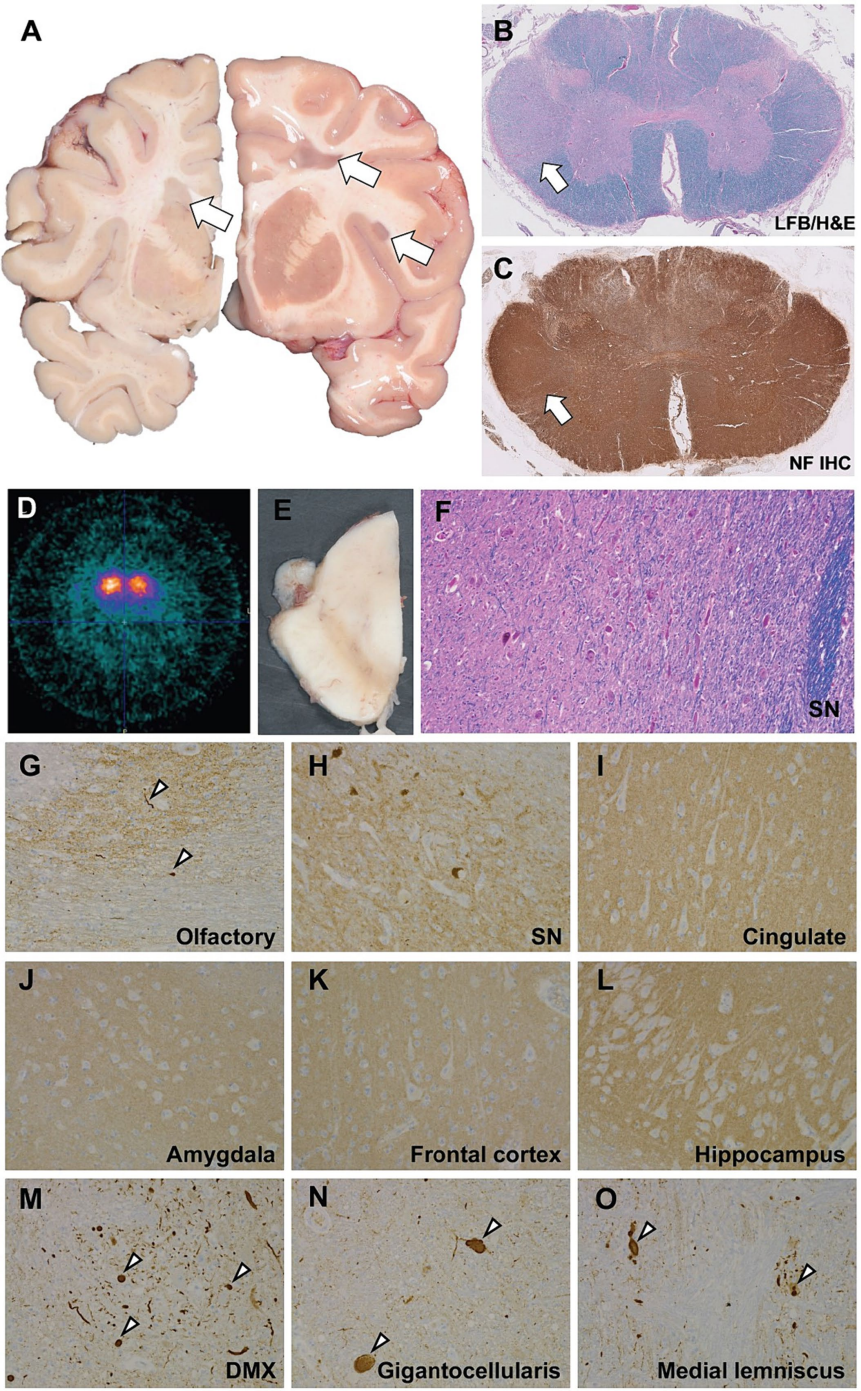
Neuropathological and neuroimaging evaluation demonstrating multiple sclerosis and brainstem degeneration in the absence of nigral Lewy bodies. **(A)** Gross coronal sections showing scattered white matter plaques (white arrows). **(B)** Luxol fast blue counterstained hematoxyoin & eosin stained (LH&E) section from the cervical spinal cord showing myelin pallor in the lateral funiculus. **(C)** Immunohistochemistry (IHC) using antisera targeting neurofilament shows intact axons consistent with a demyelinating process. **(D)** b-CIT SPECT revealed marked, symmetrically reduced uptake indicating significant loss of striatal dopamine neuronal innervation. **(E)** Gross hemisection of the midbrain demonstrating marked pallor in the substantia nigra. **(F)** High power (20x) photomicrograph of an LH&E-stained section through the midbrain showing marked loss of pigmented dopaminergic neurons and reactive gliosis. Immunohistochemistry to α-synuclein shows mild Lewy pathology (white arrowheads) in the olfactory bulb **(G)**, negative staining in the substantia nigra **(H)**, cingulate gyrus **(I)**, amygdala **(J)**, frontal cortex **(K)**, hippocampus **(L)**, and positive staining in the medulla, including the dorsal motor nucleus of the vagus (DMX; **M**), nucleus gigantocellularis **(N)**, and medial lemniscus **(O)**.

In the case with neuropathology, the participant received the diagnosis of MS in her mid-30’s when she developed left leg numbness, and this was attributed to a cervical cord lesion. During her 40’s, she experienced relapses and remissions with MRIs consistent with demyelinating plaques. Notable exacerbations included left arm weakness with loss of dexterity and, later, weakness of the left leg. Cervical spine MRI at age 67 showed abnormal signal intensity without cord expansion. Head CT at age 75 showed a hypodensity in the right frontal centrum semiovale, extending into the right periventricular region, with a similar, less prominent focus in the left centrum semiovale and subcortical white matter. She declined disease modifying treatment for MS. At age 67, she developed left arm tremor, and at age 68, she was diagnosed with PD. β-CIT SPECT revealed marked, symmetrically reduced uptake indicating significant loss of striatal dopamine neuronal innervation (Figure 1D). At age 72, examination demonstrated right hand rest tremor and bilateral action/postural tremors. In addition, there was bilateral rigidity and bradykinesia, both worse on the left. She had moderately stooped posture, required assistance with walking and tended to lose balance spontaneously. MoCA score was 21/30 (normosmic for age and sex, and her performance placed her in the best latent class, which was associated with the least severe motor decline) (9). Nigral transcranial sonography was within normal range (0.144 cm2 and 0.121 cm2) (6, 7). At age 78, she developed endometroid ovarian cancer and she died at age 80 from sepsis.

On gross neuropathological examination, there were numerous, chronic, white matter lesions bilaterally, often seen at the gray-white matter junction and periventricularly, as well as throughout the frontal, parietal, temporal and occipital lobes, consistent with demyelinating lesions (Figure 1A). In the cervical spinal cord, histological examination showed pallor and reactive gliosis in the lateral funiculus, with intact axonal neurofilament staining consistent with demyelination (Figures 1B,C). In the midbrain, there was severe pallor evident grossly; microscopically, there was loss of pigmented neurons in the substantia nigra without Lewy bodies, which was confirmed by immunohistochemistry targeting α-synuclein (Figures 1E,F,H), however mild (1+) Lewy bodies were found in the medulla and olfactory bulb (Figures 1G,M,N). Rare α-synuclein positive Lewy neurites of undetermined significance were noted in the white matter of the brainstem (Figure 1O). Additionally, the cingulate gyrus, amygdala, frontal cortex, and hippocampus were all negative for α-synuclein (Figures 1I–L).

The microscopic examination of the brain yielded various regions that were p-tau (AT8) positive including rare structures in the white matter of the substantia nigra, mild (1+) neurofibrillary tangles (NFTs) in the striatum, putamen, nucleus basalis of Meynert, amygdala, hypothalamus, and upper pons. Additionally, there were severe (3+) NFTs present in the inner and outer layers of the entorhinal area, and rare granular p-tau positive clusters and threads in the inferior frontal cortex. These findings are consistent with a Braak stage of III (B2).

Phospho-TDP-43 proteinopathy was negative in the olfactory bulb, midbrain at level of red nucleus, and all levels of the spinal cord including the cauda equina.

The second participant with *LRRK2* PD was diagnosed with MS at age 45 after the onset of horizontal diplopia. At age 48 she experienced bilateral lower extremity weakness and was treated with interferon beta-1a but discontinued due to side effects. She was switched to glatiramer acetate and stayed on this medication for at least 8 years, but did not take it consistently. Clinical features in her case were also consistent with a relapsing–remitting phenotype. At age 66, she developed stooped posture and difficulty with gait. She was diagnosed with PD and started on carbidopa-levodopa with improvement. MRI at age 75 demonstrated elongated, flame-shaped T2 hyperintense lesions perpendicular to the lateral ventricle wall as well as peri-colossally and at the parietal-occipital junction. Age-related atrophy of the bilateral cerebral hemispheres was also noted.

## Discussion

The higher rate of MS preceding PD, albeit an uncommon occurrence, supports that MS may be part of the phenotypic spectrum of *LRRK2* GS019S carriage, and provides further support for immune relation in *LRRK2*. The numerous, white matter lesions seen on neuropathology in one of the participants with MS and *LRRK2* PD, and on MRI in the other, together with nigral cell loss in the first case, substantiate a chronic, demyelinating disorder separate from, and preceding, PD. The development of MS prior to PD in individuals with the G2019S variant is not altogether surprising as *LRRK2* plays an important role in a variety of immune responses and is found within monocytes, macrophages, B lymphocytes and other immune cells [Reviewed by Zhang et al. (10)]. Several studies have shown that cells with this gain of function mutation (increased kinase activity) have exaggerated pro-inflammatory responses, including heightened release of cytokines (11, 12). Correspondingly, associations between *LRRK2* carrier status and other immune conditions, including Inflammatory Bowel Disease (IBD)/Crohn’s Disease, have been raised (13–15). Genome wide associations have found that *LRRK2* G2019S mutations are a risk factor for both Crohn’s disease (CD) and PD, with inflammatory processes proposed as common mechanistic pathways (15). Given these established links, it is worth considering that impairments in *LRRK2* G2019S may create the conditions whereby immune dysregulation underlies a subtype of neurological disease with features of both MS and PD.

Increased prevalence of autoimmune diseases relative to other PD groups was found in a Norwegian sample of 100 *LRRK2* carriers, and included three individuals (3%) diagnosed with MS (2). In roughly the same geographic location, overall MS prevalence among the local population has been estimated at 0.16%, suggesting that *LRRK2* carrier status may indeed confer an increased risk of developing MS (16). While our sample prevalence was smaller (1.4%), these results are highly suggestive of an association between MS and *LRRK2* PD that is not attributable to chance.

The presented neuropathological findings suggest that homozygous *LRRK2* mutation carriers do not have greater PD severity than heterozygotes. These results also highlight the absence of Lewy body pathology in a significant subset (17). Though a putaminal demyelinating lesion was seen on neuropathological examination, it is unlikely that this unilateral lesion was the cause of this participant’s bilateral PD symptoms. In addition, most MS patients who have basal ganglia or midbrain lesions do not develop signs of PD and parkinsonism is quite rare in MS patients overall (18).

Among the over 30 cases of homozygous *LRRK2* mutations reported to date, most are biallelic G2019S variants (3, 4, 19–22). The phenotype is similar in individuals carrying either one or two *LRRK2* G2019S mutations and, in general, both homozygous and heterozygous mutations are associated with low rates of cognitive impairment and psychiatric co-morbidity. PD in *LRRK2* homozygous carriers is not thought to be more penetrant than in heterozygous carriers and there is no evidence to suggest more severe clinical manifestations (3, 19–22). In line with these observations, our homozygous participant presented with symptoms that were, overall, consistent with relatively mild and slowly progressive Parkinson disease.

Lewy bodies, long-considered to be distinctive features of PD-associated neurodegeneration, are absent in a significant portion of *LRRK2* cases. Among 37 autopsy *LRRK2* PD cases, only 17 had Lewy bodies with Lewy body pathology found more frequently in G2019S carriers than in other *LRRK2* variants (17). Though motor features were similar among *LRRK2* PD cases with and without Lewy body pathology, non-motor features, including orthostatic hypotension, anxiety and cognitive impairment, were more frequent in the Lewy body group (17). Of interest, there was no nigral hyperechogenicity on transcranial sonography.

While olfaction has not been consistently reported in PD autopsy cases, absence of olfactory loss, and its potential association with isolated nigral degeneration, is of great interest. We have previously reported on two clusters of olfactory performance in G2019S *LRRK2* PD: in the worse-performing cluster, PD age of onset was earlier and motor decline was faster (9). We, therefore, hypothesized that the cluster with better olfaction may show isolated nigral degeneration. While larger clinical-autopsy studies are necessary to further assess this correlation, these results raise the question as to whether excellent olfaction may be a clinical surrogate for absence of synuclein pathology. This is supported by recent CSF synuclein seeding assay studies demonstrating lower frequency of SSA abnormalities in normosmic *LRRK2* PD (23).

Our participant’s significant bilateral nigral degeneration, without substantial Lewy Body pathology, extends the *LRRK2* G2019S neuropathologic phenotype to suggest that the absence of Lewy body deposition may occur even in biallelic G2019S carriers. A prior report of two homozygous R1441 mutation carriers also showed prominent nigral degeneration without alpha-synuclein pathology (24). In a separate neuropathological analysis of 10 *LRRK2* G2019S carriers (two homozygous and eight heterozygous), only half of the examined samples had alpha-synuclein pathology; unfortunately, the homozygous cases were not separately delineated (4).

Late in life this participant developed endometrial cancer. A possible association between *LRRK2* variants and non-skin cancers has also been reported, though the mechanisms of the link remains incompletely understood (25).

This study bolsters a hypothesized link between immunologic disease and *LRRK2* PD, and raises important questions about shared immune pathophysiology contributing to disease development. It also highlights the potential for personalized approaches through the lens of the pathologic heterogeneity of *LRRK2* PD. In particular, evidence (or lack thereof) of microscopic Lewy body pathology, through synuclein or surrogates for pathology, may guide choice of anti-synuclein agents. Thus, our results emphasize the importance of continuing to work toward a clear delineation of PD subtypes, as therapeutic trials focused on groups with shared causality are far more likely to succeed. In addition, neuropathological findings support that homozygous *LRRK2* mutations are not associated with worse brain pathology than heterozygote *LRRK2* mutations, though we cannot exclude the possibility that, in this case, *LRRK2* homozygosity may have contributed to the development of MS. Our findings suggest the need to investigate concurrent immune and inflammatory diseases in Parkinson’s disease, and especially in *LRRK2* PD.

## Data availability statement

The original contributions presented in the study are included in the article/supplementary material, further inquiries can be directed to the corresponding authors.

## Ethics statement

Ethical review and approval was not required for the study on human participants in accordance with the local legislation and institutional requirements. The participants provided their written informed consent to participate in this study. Written informed consent was obtained from the deceased patient’s family for publication of the details of their medical case and any accompanying images.

## Author contributions

AW: Conceptualization, Data curation, Formal analysis, Funding acquisition, Investigation, Methodology, Project administration, Resources, Software, Supervision, Validation, Visualization, Writing – original draft, Writing – review & editing. RO: Writing – original draft, Writing – review & editing. DeR: Writing – original draft, Writing – review & editing. AC: Writing – original draft, Writing – review & editing. ET: Writing – original draft, Writing – review & editing. KL: Writing – original draft, Writing – review & editing. DaR: Writing – original draft, Writing – review & editing. SB: Writing – original draft, Writing – review & editing. JC: Writing – original draft, Writing – review & editing. RS-P: Writing – original draft, Writing – review & editing.
